# Culicidae Fauna (Diptera: Culicomorpha) of the Municipality of Mazagão, Amapá, in the Brazilian Amazon

**DOI:** 10.3390/insects16101036

**Published:** 2025-10-09

**Authors:** Rafael Espíndola do Nascimento, Daniel Damous Dias, Bruna Lais Sena do Nascimento, Tiago Silva da Costa, Raimundo Nonato Picanço Souto, Livia Medeiros Neves Casseb, Joaquim Pinto Nunes Neto, Valeria Lima Carvalho

**Affiliations:** 1 Graduate Program in Virology, Evandro Chagas Institute, Ananindeua 67030-000, PA, Brazil; rafael.nascimento@unifap.br; 2 Microbiology and Immunology Laboratory, Ministry of Education, Federal University of Amapá, Macapá 68903-419, AP, Brazil; 3 Graduate Program in Parasitic Biology in the Amazon, Center for Biological and Health Sciences, State University of Para, Belem 66095-663, PA, Brazil; damous1994@gmail.com (D.D.D.); joaquimneto@iec.gov.br (J.P.N.N.); 4 Department of Arbovirology and Hemorrhagic Fevers, Evandro Chagas Institute, Secretariat of Health and Environment Surveillance, Ministry of Health, Ananindeua 67030-000, PA, Brazil; brunanascimento@iec.gov.br (B.L.S.d.N.); liviacasseb@iec.gov.br (L.M.N.C.); 5 Arthropod Laboratory, Ministry of Education, Federal University of Amapá, Macapá 68903-419, AP, Brazil; tiago_sc@hotmail.com (T.S.d.C.); rnpsouto@unifap.br (R.N.P.S.)

**Keywords:** mosquitoes, arbovirus, entomofauna, seasonal dynamics

## Abstract

**Simple Summary:**

The Amazon biome encompasses the largest hydrographic basin in the world, high humidity, extensive forests, and remarkable biodiversity. However, it has been experiencing deforestation driven by human activity. These changes directly impact the region’s climate, influencing mosquitoes of the Culicidae family, which are vectors of major arthropod-borne diseases, such as *Orthoflavivirus dengue*, *Alphavirus chikungunya*, *Orthobunyavirus oropoucheense*, and *Orthoflavivirus zikaense*. This study aimed to investigate the fauna of Culicidae in the rural zone of the district of Mazagão Velho, within the municipality of Mazagão, in the state of Amapá, Brazil. Three excursions were carried out between 2023 and 2024. The collection methods used were human attraction and CDC traps, in the ground and using canopy modalities. A total of 3500 specimens were collected across the three seasonal periods: rainy with 1079 specimens, intermediary with 2172, and dry with 249. The genus *Culex* exhibited the highest abundance, followed by the genus *Coquillettidia*. The presence of epidemiologically relevant species involved in arbovirus transmission highlights the need for further entomofaunal studies in the state and underscores the potential risk to public health.

**Abstract:**

The Amazon hosts one of the richest diversities of mosquitoes in the family Culicidae, which are key both as arbovirus vectors and as environmental bioindicators. However, the state of Amapá remains poorly studied regarding its mosquito fauna. This study aimed to characterize the diversity and seasonal composition of Culicidae in the municipality of Mazagão, Eastern Amazon, within a rural landscape influenced by human activity and extreme climatic events. Three sampling campaigns were conducted between 2023 and 2024, covering rainy, intermediary, and dry periods. Mosquitoes were collected using Protected Human Attraction (PHA) and CDC light traps at both ground and canopy strata. A total of 3500 specimens were obtained, representing 38 species across 15 genera. The intermediary period yielded the highest abundance and richness, whereas the dry season presented very low diversity, probably because of severe drought and forest fires. Dominant species included *Coquillettidia* (*Rhy.*) *venezuelensis*, *Cq. albicosta*, and *Mansonia titillans*. There were significant differences in community diversity between dry and wetter periods, underscoring the strong role of seasonality in shaping mosquito populations. These findings represent the entomofaunistic survey of the region, contributing to biodiversity knowledge and highlighting potential public health risks, thus reinforcing the need for continuous entomological monitoring.

## 1. Introduction

The Amazon biome encompasses the largest hydrographic basin in the world, the Amazon River basin, characterized by well-defined dry and rainy seasons, high humidity, extensive forests, and remarkable biodiversity, conditions that are highly favorable for the mosquito life cycle. It covers an area of 7.6 million km^2^ in South America, of which more than half lies within Brazilian territory, accounting for approximately 45% of the country’s land area. However, alterations driven by unsustainable land use and climate change have been altering temperature and rainfall patterns, impacting the biome, modifying ecological relationships, and exerting selective pressures that lead to changes in the life cycle and habitats of animal species such as mosquitoes [1,2,3,4].

Insects belonging to the family Culicidae Meigen, 1818, are particularly sensitive to climate change and alterations in the landscape; furthermore, they are closely related to the transmission of several diseases, such as arboviruses, malaria, and filariasis. Therefore, understanding their diversity, spatial distribution, and the environmental factors that may influence mosquito dynamics is highly relevant to epidemiology within the One Health framework [3,4].

The Brazilian Amazon has been experiencing deforestation driven by human activity. Approximately 20% of the original forest cover has been lost, altering its diverse ecosystem, affecting temperature, and impacting the health of local human populations. In 2024, the deforested area reached 5816 km^2^, primarily due to forest fires [5]. The Amazon fauna harbors a wide diversity of known pathogens, as well as potential unknown pathogens, as exemplified by the recent epidemic caused by the *Orthobunyavirus oropoucheense* (OROV), the etiological agent of Oropouche fever, which is endemic to the Amazon region but has spread across the country and other Latin American and Caribbean countries in 2024 [6,7]. Alterations of such magnitude can lead to changes in mosquito composition, modify their habitats, and may facilitate the spread of arthropod-borne diseases [6,8].

The first recorded epidemic likely caused by an arbovirus in Brazil occurred in 1685 in Recife, Pernambuco. Based on historical accounts and clinical symptoms, it is retrospectively attributed to Yellow Fever Virus (YFV), currently classified as *Orthoflavivirus flavi* [9]. Since then, several arboviruses have circulated in the country. In the Brazilian Amazon region, 198 arboviruses had been isolated by 2010, of which 104 were confirmed as novel to science, and 34 have the capacity to infect humans [10]. Among them are *Orthoflavivirus denguei* (DENV), *Alphavirus mayaro* (MAYV), OROV, YFV, *Orthoflavivirus nilense* (WNV), and many others [8].

Understanding the entomofauna of Culicidae is relevant not only from an epidemiological perspective but also as a forest bioindicator. Differences in diversity and abundance, behavioral changes in species, or even the absence of a given taxon are observed characteristics that may indicate disturbances in the environment [11]. For example, the abundance of *Aedes scapularis* and mosquitoes of the tribe Mansoniini serve as indicators of human-induced environmental alterations, since they are seldom found in preserved habitats [12,13].

Currently, approximately 3700 species of Culicidae are recognized worldwide, of which around 500 occur in Brazil [14,15]. Species of the Culicidae family play a crucial role in the vast, diverse, and complex Amazonian ecosystem [2]. Entomofaunistic studies conducted in the Amazon region have already recorded about 320 species, although many others are estimated to remain undocumented [16].

The state of Amapá is located in the eastern Amazon, with its capital city crossed by the Equator at the mouth of the Amazon River, and it exhibits endemic circulation of several arboviruses. From 2000 to 2023, there were 40,760 confirmed cases of DENV. Between 2015 and 2023, ZIKV caused 1784 cases in the state [17]. OROV triggered an epidemic in 2023 that originated in the Amazon [18] and spread across Brazil, resulting in 125 cases in Amapá in 2024 [19]. Amapá was also the entry point of the Asian genotype of CHIKV, due to its position as a border region between Brazil and French Guiana, leading to 3855 cases between 2014 and 2023 [17,20].

Few studies have investigated the Culicidae fauna in Amapá. The species list from the Culicidae collection of the Entomological Collection of the Scientific and Technological Research Institute of the State of Amapá (IEPA) includes a total of 66 species and 9 morphotypes, distributed across the subfamilies Anophelinae and Culicinae; the tribes Aedeomyiini, Aedini, Culicini, Mansoniini, Orthopodomyiini, Sabethini, Uranotaeniini, and Toxorhynchitini; and the genera *Anopheles* Meigen, *Aedes* Meigen, *Aedeomyia* Theobald, *Haemagogus* Williston, *Coquilletidia* Dyar, *Culex* Linnaeus, *Johnbelkinia* Zavortink, *Psorophora* Robineau-Desvoidy, *Mansonia* Blanchard, *Orthopodomyia* Theobald, *Limatus* Theobald, *Runchomyia* Theobald, *Sabethes* Robineau-Desvoidy, *Wyeomyia* Theobald, *Trichoprosopon* Theobald, *Uranotaenia* Lynch Arribálzaga, and *Toxorhynchites* Theobald [21].

Understanding how mosquitoes respond to factors such as seasonal change, land use changes, or climate change is essential for predicting risks of arbovirus transmission. The present study was conducted in the municipality of Mazagão, state of Amapá, in the Brazilian Amazon region. This study sought to identify which mosquito species circulate in the area, highlighting the potential vectors of arboviruses. The findings may support public health and economic development policies while integrating these objectives with environmental conservation.

## 2. Materials and Methods

### 2.1. Ethical Aspects

Authorization for the collection of mosquito specimens in the rural area of the municipality of Mazagão, Amapá, was granted under permit number 87915 by the Biodiversity Authorization and Information System (SISBIO) of the Chico Mendes Institute for Biodiversity Conservation (ICMBio) (https://sicae.sisicmbio.icmbio.gov.br/, accessed on 23 September 2025).

### 2.2. Study Area

**Figure 1:**
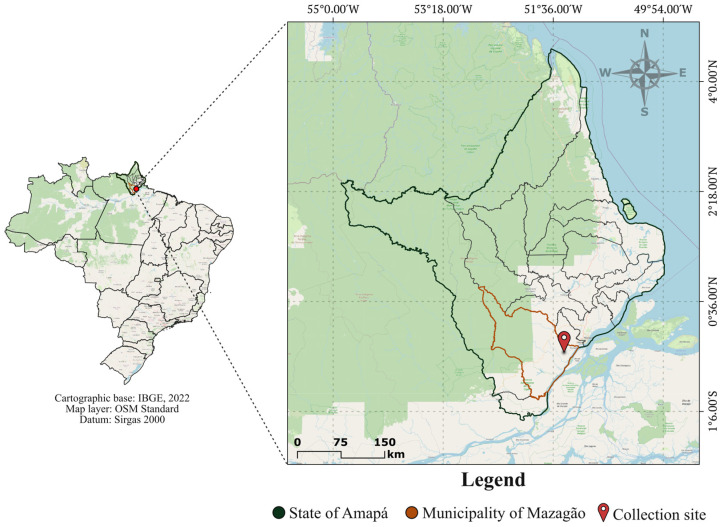
Location map of the municipality of Mazagão, showing the mosquito sampling site. This figure was created using QGIS v.3.22 (https://qgis.org/en/site/, accessed on 5 August 2025), based on cartographic data from the Brazilian Institute of Geography and Statistics (IBGE, 2022; https://www.ibge.gov.br/, accessed on 5 August 2025) and raster layers from the OpenStreetMap project (https://www.openstreetmap.org/#map=4/-14.39/-57.39, accessed on 5 August 2025). On the left, the map of Brazil is shown; the right panel highlights the state of Amapá (in green), the municipality of Mazagão (in orange), and the sampling site (marked in red).

This was an eco-epidemiological study on Culicidae fauna carried out in the northern part of the Eastern Amazon, in Brazil, during July and November 2023 and April 2024. The study area is located in the rural zone of the district of Mazagão Velho, within the municipality of Mazagão, in the state of Amapá, Brazil (Figure 1). The vegetation is native and characterized by a tropical climate and fauna intrinsic to this environment. The region has high temperatures and humidity throughout the year, with a rainy season from November to April and a dry season from May to October (https://en.climate-data.org/, accessed on 23 September 2025).

The district of Mazagão Velho is rich in history and traditions, annually attracting numerous tourists. It is notable for the traditional São Tiago festival, its natural attractions such as river bathing, and its typical cuisine [22]. However, the intense flow of visitors increases the risk of arbovirus infection, and, upon returning to their places of residence, tourists may contribute to the introduction and dissemination of these viruses in urban areas.

The collections were carried out at Vila Maranhão Farm, located 4 km from the center of the district of Mazagão Velho and 25 km from the municipal seat of Mazagão (0°10′52.01″ S; 51°25′57.04″ W), at an altitude of 9 m. The collection place was close to an area of small-scale cassava cultivation.

The native vegetation is predominantly composed of secondary forest, with large trees averaging 15 m in height, and a rich, abundant fauna, with frequent sightings of birds, monkeys, squirrels, and rodents. Approximately 100 m from the mosquito sampling site, there was a lake and its floodplain vegetation—an environmental factor providing ideal conditions for the maintenance of some mosquito life cycles and other animals.

### 2.3. Entomological Collection

Three five-day sampling campaigns were carried out over 10 months, totaling 15 sampling days: rainy season (18–22 April 2024), dry season (13–17 November 2023), and intermediary season (21–25 July 2023). The collection methods included Protected Human Attraction (PHA) and CDC-type light traps in both ground and canopy strata, following the recommendations of the Brazilian Ministry of Health [23].

#### 2.3.1. Protected Human Attraction (PHA) Method

The Protected Human Attraction (PHA) method is designed to collect adult female mosquitoes with anthropophilic behavior. This technique involves two professionals serving as human baits, fully protected by boots, jeans, hooded jackets, helmets, and ski masks. One collector is positioned in the tree canopy at a height of 15 m, while the other remains at ground level. Field collections are conducted for five hours per day over a period of 15 days. The same individuals were involved in all three collection periods without alternation between tree canopy and ground-level positions. The PHA method targets diurnal mosquitoes attracted by human odor, body heat, and sweat while seeking blood meals. During each expedition, mosquitoes are consistently collected between 7:00 a.m. and 12:00 p.m. using hand nets and oral aspirators.

#### 2.3.2. Collection with CDC-Type Traps

CDC light traps [24] were used to collect arthropods with positive phototaxis during the crepuscular/nocturnal period, being set up at 6:00 p.m. and removed at 7:00 a.m. Upon approaching the trap, mosquitoes were immediately drawn in by a fan and retained in a temporary storage compartment. In the present study, two CDC light traps were used at different strata: one at ground level and another in the tree canopy, positioned at 13 m in height, operating continuously throughout each campaign. Sampling totaled 15 days, considering the three campaigns conducted. Specimens were placed in cryotubes properly labeled with date, collection method, and stratum, and subsequently stored in liquid nitrogen at −196 °C for taxonomic identification.

#### 2.3.3. Taxonomic Identification

Taxonomic identification was performed through the observation of external morphology of specimens, using a cold table (Eletrohospitalar, Brasília, Federal District, Brazil) adjusted to approximately −38 °C, and Zeiss Stemi 2000-C stereomicroscopes (Carl Zeiss, Oberkochen, Germany). Dichotomous keys by Consoli and Oliveira (1994) [25], Galindo et al., (1954) [26], Sallum and Forattini (1996) [27], Sá et al., (2020) [28], Lane (1953) [29], and Forattini (2002) [30] were used, and abbreviations for genera and subgenera followed the recommendations proposed by Reinert (2009) [31]. After identification, specimens were grouped into lots, placed in 2 mL Eppendorf tubes containing individuals of the same species, and labeled with a unique alphanumeric registration code for arthropod samples.

### 2.4. Faunal Analysis

The normal distribution of the variables was assessed using the Shapiro–Wilk test. Homogeneity of variances was verified with Levene’s test, while outlier detection was performed through boxplot analysis and the quartile and interquartile range (IQR) method. The nonparametric Kruskal–Wallis analysis of variance, followed by Dunn’s post hoc test with Bonferroni correction for multiple comparisons, was used to determine whether there were differences in the observed diversity among the studied periods. *p*-values < 0.05 were considered statistically significant.

#### 2.4.1. Species Dominance

Dominance categories were defined according to the classification proposed by Friebe (1983) [32] and calculated using the formula D% = (i/t) × 100, where *i* corresponds to the total number of individuals of a species and *t* to the total number of individuals collected. According to this criterion, the categories are eudominant (D > 10%), dominant (5% < D ≤ 10%), subdominant (2% < D ≤ 5%), accessory (1% < D ≤ 2%), and rare (D ≤ 1%).

#### 2.4.2. Diversity Analysis

Diversity was estimated using Hill numbers [33], where “q” represents the order of diversity (qD), determining the relative influence of common and rare species in the estimation. Three orders were evaluated: q = 0, corresponding to species richness; q = 1, equivalent to the exponential of Shannon’s entropy index (H), which integrates richness and evenness while giving greater weight to more abundant species; and q = 2, corresponding to the inverse of Simpson’s concentration index (D), which reflects dominance and is inversely proportional to diversity—higher values of D indicate lower diversity, as they increase the probability that individuals belong to the same species. The indices were calculated using the *hillR* package v.0.5.2 [34].

Sampling effort was assessed using the R package *iNEXT* v.3.0.1 [35], which generated rarefaction and extrapolation curves based on species richness and individual abundance. Interpolation curves allowed comparison of observed diversity among the analyzed periods, while extrapolation curves projected the expected richness with increasing sampling effort [36]. Estimates followed the Chao1 model [37], which uses the frequency of rare species to adjust projections. A total of 1000 bootstrap iterations were performed, ensuring statistical robustness and reliable confidence intervals. The resulting curves were interpreted to assess whether the sampling effort was sufficient to represent the true community diversity or whether additional sampling might reveal new species.

Ordination curves were generated to evaluate the distribution pattern of abundances as a function of species ranking in order to assess community structure and the degree of dominance among taxa, using an internally developed R script.

#### 2.4.3. Species Overlap Across Different Periods

Species overlap among the different seasonal periods was evaluated using a Venn diagram, designed to illustrate the number and proportion of exclusive and shared species among the rainy, intermediary, and dry periods. For this analysis, the species list recorded in each period was used to construct a presence–absence matrix. The diagram was generated with the R package *ggVennDiagram* v.1.4.4 [38]. Final images were produced using Inkscape v.1.4.2 (https://inkscape.org/, accessed on 25 July 2025).

#### 2.4.4. Environmental Variables

The average temperature (°C) and relative humidity (%) were recorded every 60 min using a calibrated digital thermo-hygrometer (Kasvi, Pinhais, Paraná, Brazil). Precipitation data, accumulated over 24 h, were obtained from the National Institute of Meteorology (INMET) (https://portal.inmet.gov.br/, accessed on 31 July 2025).

## 3. Results

A total of 3500 mosquito specimens were collected during the three campaigns across the three seasonal periods: rainy (precipitation between 0 and 11.7 mm, average temperature 27.3 °C, and average relative humidity 89.1%), dry (precipitation 0 mm, average temperature 33.9 °C, and average relative humidity 62.3%), and intermediary (precipitation between 0.2 and 6.6 mm, average temperature 28.3 °C, and average relative humidity 87.4%) (Supplementary Tables S1–S3). The PHA method collected 419 specimens, while the light attraction method using CDC traps collected 3081 specimens. The total sampling effort amounted to 270 h, comprising 75 h with PHA collections and 195 h with CDC collections. No collection was carried out on rainy days.

Of the total specimens collected, 1079 were obtained during the rainy season, 2172 during the intermediary season, and 249 during the dry season. A total of 38 mosquito species were identified, distributed across 15 genera. Abundance was highest during the intermediary season, suggesting that this period is the most favorable for mosquito reproduction and survival.

The most abundant genus was *Coquillettidia* Dyar, with 1481 individuals (40.71%), followed by *Culex* with 932 individuals (25.32%) and *Mansonia* with 560 individuals (15.39%). The ground-level CDC trap captured the largest proportion of individuals (61.21% of the total), while the species most frequently collected by the PHA method was *Coquillettidia* (*Rhy.*) *venezuelensis*, followed by *Psorophora* (*Jan.*) *ferox*.

The dominant species was *Cq.* (*Rhy.*) *venezuelensis* with 633 individuals (17.40%), followed by *Cq.* (*Rhy.*) *albicosta* with 208 individuals (5.72%) and *Ma.* (*Man.*) *titillans* with 203 individuals (5.58%), as shown in Table 1.

**Figure 2:**
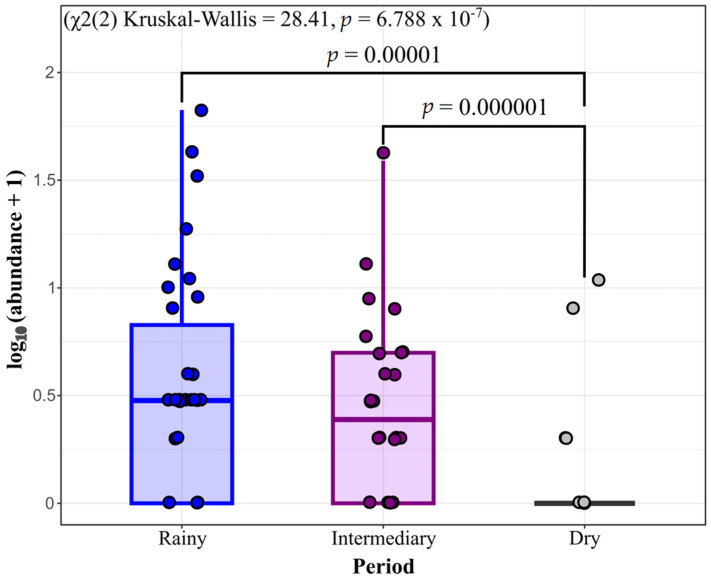
Boxplots comparing the diversity of mosquito species among the periods rainy (blue, April 2024), intermediary (purple, July 2023), and dry (gray, November 2023), in Mazagão Velho, Brazil.

The variance analysis (χ^2^(2) = 28.41, *p* = 6.79 × 10^−7^) revealed significant differences in diversity among the collection periods. Dunn’s post hoc test showed highly significant differences between the intermediary and dry periods (*p* = 0.00001) and between the rainy and dry periods (*p* = 0.000001). In contrast, no significant difference was observed between the rainy and intermediary periods (*p* = 1.000) (Figure 2).

**Figure 3:**
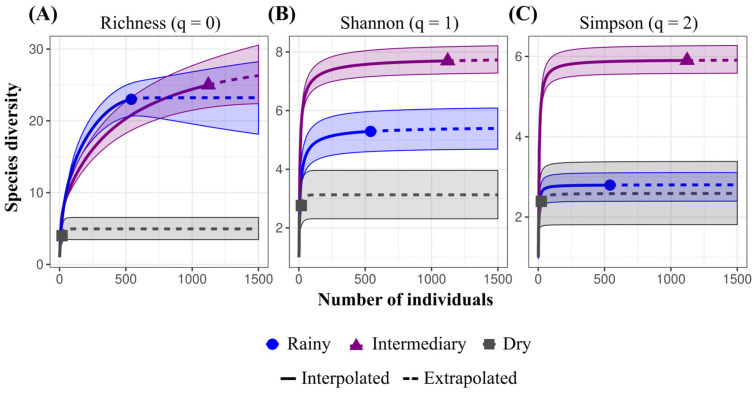
Sample effort curves for (**A**) species richness (q = 0), (**B**) Shannon diversity (q = 1), and (**C**) Simpson index (q = 2) during the rainy (blue, April 2024), intermediary (purple, July 2023), and dry (gray, November 2023) periods in Mazagão Velho, Brazil. The curves depict the increase in expected richness and effective diversity as a function of sampling effort, demonstrating seasonal variations in mosquito community composition.

The sample effort curves revealed variations in mosquito diversity across the studied periods (Figure 3). For species richness (q = 0), the rainy season showed a higher estimated number of species with effort, suggesting greater efficiency in initial detection, while the intermediary period recorded the highest observed richness (25 species). The dry period yielded only four species, with nearly flat curves, reflecting low diversity. For Shannon diversity (q = 1), the intermediary period exhibited the highest effective diversity (7.68), followed by the rainy (5.30) and dry (2.76) periods, indicating that the higher richness in the intermediary period was accompanied by a more even distribution of abundances. For Simpson diversity (q = 2), which gives greater weight to dominant species, the intermediary period again exhibited the highest diversity (5.90), followed by the rainy (2.79) and dry (2.40) periods. These results indicate that in the intermediary period, not only were more species present, but also that the community was less dominated by a few abundant taxa, suggesting a more balanced distribution of species. In contrast, both rainy and dry periods showed lower Simpson diversity, reflecting stronger dominance by particular species, even though richness was higher in the rainy period. The comparison between interpolated and extrapolated curves suggests sufficient sampling effort for the intermediary and dry periods but indicates greater uncertainty for the rainy period, particularly regarding richness, implying that additional sampling could reveal additional species.

**Figure 4:**
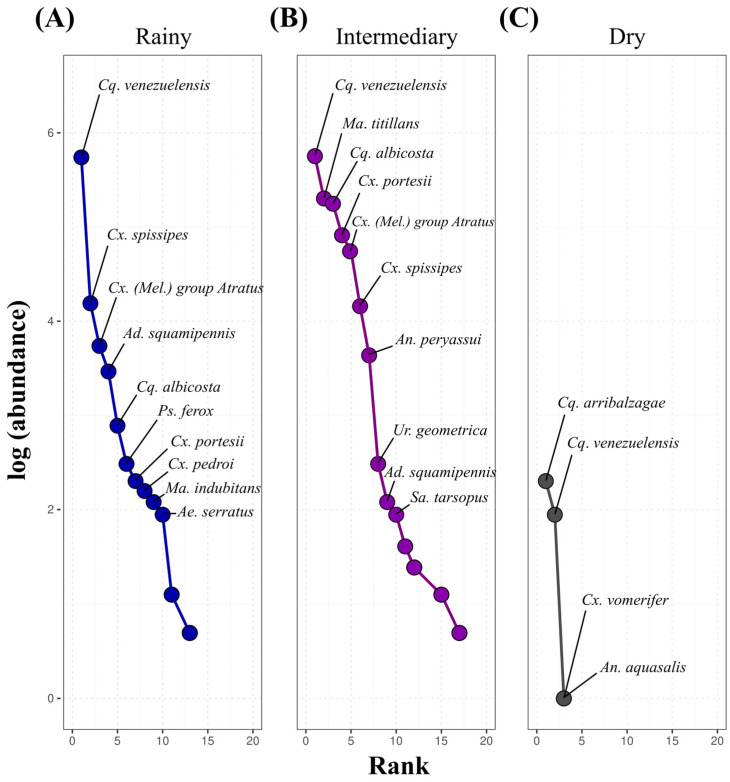
Rank-abundance curves of mosquito species during the (**A**) rainy (blue, April 2024), (**B**) intermediary (purple, July 2023), and (**C**) dry (gray, November 2023) periods in Mazagão Velho, Brazil. This figure shows the distribution of abundance (Y-axis, logarithmic scale) as a function of species rank (X-axis), ordered from the most to the least abundant in each period.

The rank-abundance curves indicated seasonal variations in mosquito community composition (Figure 4). During the intermediary period, the highest diversity and balance in abundance distribution were recorded, with *Cq. venezuelensis* and *Ma. titillans* as the most abundant species, accompanied by others with intermediary occurrence. In the rainy period, diversity was moderate, with dominance of *Cq. venezuelensis* and *Cx. spissipes*, followed by a sharp decline in the abundances of other species. In the dry period, the community was less diverse, with a strong predominance of *Cq. arribalzagae* and *Cq. venezuelensis* and overall reduced abundance, evidencing the impact of adverse environmental conditions on species richness and distribution.

**Figure 5:**
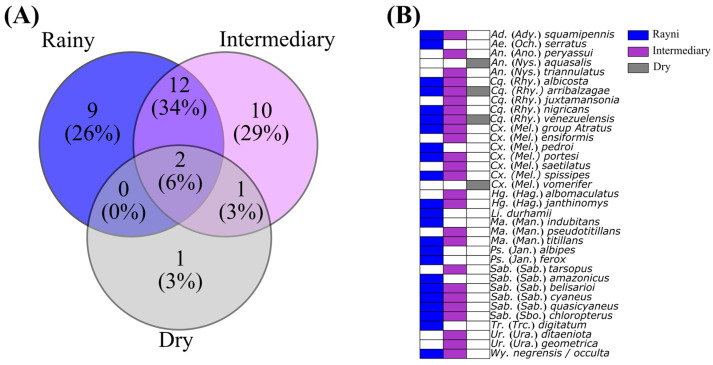
Venn diagram compiling the distribution of species richness (**A**) and overlap of mosquito species across the rainy (blue, April 2024), intermediary (purple, July 2023), and dry (gray, November 2023) periods in Mazagão Velho, Brazil (**B**).

The composition of mosquito communities revealed seasonal variation (Figure 5). The rainy period exhibited nine exclusive species (26% of the total), while the intermediary period had ten exclusive species (29%), indicating high diversity and differentiation between these phases. The dry period showed only one exclusive species (3%) and low overlap with the other periods. The greatest similarity was observed between the rainy and intermediary periods, which shared 12 species (34%), whereas only two species (6%) occurred across all periods.

## 4. Discussion

The Amazon region is one of the most important ecosystems on the planet due to its vast biodiversity. The region provides ideal environmental conditions for the maintenance of arboviruses, supported by complex cycles involving these hematophagous arthropods as vectors and vertebrates as hosts [39].

In this study, 38 species distributed across 15 genera were identified. This is the first survey to include collections from Vila Maranhão farm across three distinct periods: rainy, dry, and transitional. Previous studies conducted in the state of Amapá provide important contributions to the understanding of Culicidae diversity in the region, allowing for better contextualization of the results obtained in the present work. For example, Silva [40] investigated this diversity in a biological reserve also in the Amapá state during both the less rainy and rainy periods, between October 2016 and March 2017, with a sampling effort of 11 days per month. In that study, 49 species distributed across 15 genera were identified, with greater abundance and richness recorded during the rainy period. Similarly, Souto [41] carried out collections exclusively during the rainy season, also in a forested area of Amapá, recording 35 species distributed across 13 genera. In a broader context, Cerqueira (1961) [42] conducted a pioneering survey on the distribution of culicids in the Amazon, using samples obtained from the former National Yellow Fever Service and from collections of the National Institute for Amazonian Research. This study cataloged 218 species throughout the Amazon region, of which 60 species, belonging to 13 genera, were recorded in seven localities of Amapá, representing to this day the most comprehensive survey ever conducted for the state.

In comparison, faunistic surveys conducted in other areas of the Brazilian Amazon yielded similar results. In the state of Pará, Dias [43] investigated the Culicidae fauna in a forest fragment embedded in an urban matrix, conducting collections in April and May (rainy season) and August and September (dry season) of 2023. The sampling effort employed the protected human attraction (PHA) and CDC light trap methods. This study recorded 34 species distributed across 10 genera, with higher abundance and richness observed during the rainy season. Similarly, Reis et al. [3] carried out a survey in a protected environmental area also in Pará, where 56 species distributed across 13 genera were recorded. Collections were conducted in two campaigns in 2023, the first during the rainy season in April and May, and the second during the dry season in August and September. Using the CDC and PHA capture methods, the study also found greater species richness and abundance during the rainy season, highlighting the influence of seasonality on the composition of the culicid fauna.

In contrast with these findings, the present study in Amapá identified the transitional period between the rainy and dry seasons as the time of highest culicid abundance and species diversity, followed by the rainy and then the dry seasons. This divergence may be attributed to the unique ecological and climatic characteristics of the region, where fluctuating environmental conditions during the transitional period create favorable microhabitats and breeding sites that support a richer and denser mosquito fauna. Climatic seasonality strongly influences the mosquito life cycle, affecting breeding site availability, larval development rates, and adult survival, thereby shaping population dynamics over time [44].

The vast biodiversity of the Amazon region is evident in the different species that dominate across its various localities. In this study, the genera *Coquillettidia* and *Culex* were the most abundant, while *Cq.* (*Rhy.*) *venezuelensis*, *Cq.* (*Rhy.*) *albicosta*, and *Ma.* (*Man.*) *titillans* were the dominant species, a result similar to those reported by Reis (2025) with 10 days of sampling efforts [3], Silva with 121 days (2008) [40], and Souto [41] with six, also conducted in the Eastern Amazon with CDC and PHA methods. In the Western Amazon, Fé [45] reported *Mansonia titillans* and *Mansonia humeralis* as the most frequent species over 25 days of sampling with CDC, PHA, and larvitrap methods, whereas Almeida [46] found *Culex urichii* as the most abundant, followed by *Aedes albopictus,* with eight days using larvitraps as the only method. Another study conducted in a rural area of Amazonas state reported *Ps. amazonica* and *Hg. janthinomys* as dominant species [47] over 36 days with PHA and larvitrap methods. Environmental conditions, sampling methods, selective pressures, and species adaptability are factors that may influence species dominance and abundance.

Regarding the sampling methods, the analysis across the three climatic periods revealed clear and consistent patterns of efficiency. Over the entire study, CDC light traps accounted for 3081 specimens (88.03% of all mosquitoes collected), whereas PHA yielded 419 specimens. Seasonal totals followed a similar trend, with CDC collections reaching 955 individuals in the rainy period (CDC = 955; PHA = 124), 1888 in the intermediary period (CDC = 1888; PHA = 284), and 238 in the dry period (CDC = 238; PHA = 11). Both methods showed their highest yields during the intermediary period, reflecting an overall increase in mosquito abundance at this time.

Some taxa were predominantly sampled by CDC traps across all seasons, including *Cq. venezuelensis* (605 individuals, 96% by CDC), *Culex* (*Mel.*) spp. (421 individuals, 99% by CDC), and *Mansonia* (*Man.*) spp. (345 individuals, 99% by CDC). PHA, in turn, proved particularly important for detecting anthropophilic taxa such as *Cq.* (*Rhy.*) spp. and *Ma. titillans*, which, although collected in smaller numbers, showed proportionally higher representation in human-baited collections during the rainy and intermediary periods.

When comparing the two sampling approaches, clear differences also emerged in terms of taxonomic representation. Although several taxa could not be identified to the species level, the analysis considered all units identified either as species or morphospecies. CDC light traps were markedly more effective in terms of abundance, collecting the vast majority of individuals, while PHA, despite collecting fewer specimens, recorded a higher richness of taxa, encompassing both identified species and morphospecies. This pattern highlights that CDC traps are superior for collecting large numbers of mosquitoes, whereas PHA contributes more strongly to detecting a broader array of taxonomic units, thereby increasing the overall representation of the local mosquito fauna. Previous studies conducted in the Amazon region have reported findings consistent with those of the present study. For example, a survey along the Nhamundá and Abacaxis rivers in the state of Amazonas found that CDC light traps (with ultraviolet light) accounted for 53% of the species sampled and, together with Shannon-type traps, were responsible for 90% of mosquito collections [48]. Similarly, along the Padauari River, Amazonas, it was reported that CDC light traps yielded the highest number of specimens compared to other collection methods, including human bait in both domestic and natural environments [49].

In contrast, other studies have shown that human-baited collections can outperform CDC traps in both abundance and species richness, including those conducted in the municipality of Caxiuanã, state of Pará [50]; by Farias (2021) in an insular region of Belém [51]; by Dias (2024) in a secondary forest fragment within an urban area also in Belém, Pará [40]; and by Reis et al. (2025) in an environmental protection area in the municipality of Ananindeua, Pará [3]. Similarly, in the state of São Paulo, researchers compared different collection methods and found that the hand-net (PHA) yielded the highest species diversity and abundance in both canopy and ground strata, outperforming CO_2_-baited traps and BG-Lure^®^, and highlighted its effectiveness for collecting diurnal mosquitoes [52,53].

It is important to note, however, that each method exhibits a degree of selectivity for particular ecological traits [54,55]. CDC traps, which rely on light as an attractant, are especially suited for broad surveys and for collecting a greater diversity of species, particularly those with nocturnal or crepuscular habits [56]. In contrast, PHA selectively targets species that are more attracted to humans and therefore of greater epidemiological significance, even when present at lower densities. This complementarity highlights the value of using both approaches to obtain a more comprehensive picture of mosquito community structure and to detect species of medical importance that might be underrepresented if only a single sampling method were employed [55].

In the present study, seasonal analyses further revealed that mosquito abundance during the dry period differed significantly from that of the rainy and intermediary seasons. This pattern reflects the biology of mosquitoes, which are holometabolous insects whose complete metamorphosis includes the egg, larval, pupal, and adult stages, where the first three phases depend on aquatic environments for development [57]. During the dry season in the Amazon, the availability of water in natural and artificial breeding sites decreases considerably, limiting reproductive success and the survival of immature stages. Previous studies indicate that mosquito species diversity and population abundance tend to be higher during rainy periods, when greater water availability allows for the formation of multiple aquatic microhabitats, favoring egg hatching and larval development [3,43,58]. In addition, environmental factors associated with rainfall, such as increased relative humidity and thermal stabilization, may positively influence adult activity, dispersal, and feeding, thereby contributing to population maintenance [25,30]. This seasonal pattern has also been observed in different regions of the Amazon and in other tropical biomes, reinforcing the importance of rainfall regime as a key determinant of population dynamics and community structure in culicids [59,60].

The abundance rank curves highlight the adaptive capacity of the genera *Coquillettidia*, *Culex*, and *Mansonia* in the sampling environment of this study, all of which are of medical relevance as they include species that act as arbovirus vectors [30]. The proximity to the lake and its floodplain vegetation with macrophytes creates favorable conditions for mosquitoes of the tribe Mansoniini proliferation [25].

The species *Cq. venezuelensis* was ubiquitous across the three studied periods, showing high abundance with 633 individuals (18.09%), and was classified as eudominant within the community. The genus *Coquillettidia* is widely distributed in tropical and subtropical regions, particularly in the Americas, Asia, and Africa, and is adapted to a variety of aquatic habitats [25,30]. This species has high medical relevance due to its feeding behavior, characterized by aggressive biting and marked anthropophily. It is of considerable epidemiological importance, as it has been found naturally infected with several arboviruses, including WNV [59], *Arurhavirus aruac*, *Bimiti virus* [61], *Orthobunyavirus gamboaense* [62], MAYV [25], *Phlebovirus itaporangaense*, *Bussuquara virus*, and *Moju virus* [63]. Studies also suggest that *Cq. venezuelensis* may act as a secondary vector of OROV, potentially contributing to the maintenance and circulation of these agents in sylvatic cycles [64,65]. Its consistent presence and high abundance across the different sampling periods reinforce the ecological and epidemiological importance of this species in Amazonian ecosystems, highlighting its relevant role both in mosquito population dynamics and in the transmission of arboviruses in the region.

The species *Cq. albicosta* was the second most abundant, classified as dominant, with 208 specimens (5.94%) collected mainly during the rainy and intermediary periods. Although few studies have addressed its ecology and distribution, this species has been recorded primarily in South American countries [66]. *Cq. albicosta* also has medical relevance, having been found naturally infected with arboviruses, including ZIKV [67] and *Alphavirus tonate* [68] found in French Guiana. Its occurrence during periods of higher water availability may be associated with the dependence on aquatic breeding sites for larval development, reflecting seasonal patterns observed in other culicids of medical importance. These factors highlight the need for further studies to better understand their ecological and epidemiological role in Amazonian ecosystems.

Another dominant species identified in the present study was *Mansonia* (*Man.*) *titillans*, with 203 individuals (5.80%) collected. This species is widely distributed throughout the American continent, ranging from the southern United States to southern Argentina [69]. *Ma. titillans* has recognized medical importance, with records of natural infection with MAYV [70] and acting as an effective vector of *Alphavirus venezuelan* in studies conducted in Peru [71] and Venezuela [72]. In addition, investigations carried out by Beranek et al. [73] in Argentina identified, through Polymerase Chain Reaction (PCR), the presence of *Orthoflavivirus louisense* (SLEV) in pools of this species.

The genus *Culex* has a remarkable ability to adapt to a wide range of environments and is a primary vector of several arboviruses, being considered a major challenge in vector control strategies. In Brazil, 42 arboviruses belonging to this genus have already been identified [3,74]. In the present study, the species *Cx.* (*Mel.*) *portesi* and *Cx.* (*Mel.*) *spissipes* were classified as subdominant; however, when analyzing the intermediary period separately, *Cx.* (*Mel.*) *portesi* stood out as dominant, with 136 individuals collected (6.26%). This species was also the most abundant in a study conducted by Reis et al. (2025) [3] in the State of Pará, in an environment with similar characteristics, and is a recognized natural vector of *Alphavirus venezuelensis* [75]. Moreover, the genus *Culex* has been associated with the transmission of other arboviruses, such as WNV, *Orthoflavivirus japonicum* [76], *Alphavirus mucambo*, *Hapavirus mosqueiro*, *Orthobunyavirus maritubaense* [77], *Bussuquara virus*, *Orthoflavivirus ilheusense* [78], and SLEV [79].

During the dry season, the nearby lake was dry, and few specimens were collected, with a predominance of the genus *Culex*, mainly collected using the CDC light trap method. The community exhibited lower diversity, characterized by a strong predominance of *Cq. arribalzagae* and *Cq. venezuelensis*, along with a generally reduced abundance. Similarly, a study conducted in the Piratuba Lake Ecological Reserve, in Amapá, during the dry season and employing 11 sampling sites, recorded 1731 Culicidae specimens. The most abundant species were *Ma.* (*Man.*) *titillans*, *Cq.* (*Rhy.*) *venezuelensis*, *Ad. squamipennis*, *Ur.* (*Ura.*) *geometrica*, *An.* (*Nys.*) *albitarsis*, *Cq.* (*Rhy.*) *albicosta*, *Cx.* (*Cux.*) *coronator*, *Ae.* (*Och.*) *scapularis*, *Cx.* (*Cux.*) *declarator*, and *Cx.* (*Mel.*) *portesi* [40].

The historical average number of fire outbreaks in the state of Amapá during the dry season in the Amazon region (from August to November) between 1998 and 2023 is 1764 outbreaks per year. However, during the sampling period of this study, an intense drought was observed, with 2552 fire outbreaks recorded [80,81], a value significantly above the historical average. The sampling site was heavily impacted by smoke from these fires, which may have contributed to the marked reduction in mosquito diversity and abundance observed in the dry season. Furthermore, many mosquitoes collected in the CDC traps were found dead and extremely dry inside the trap cups, hampering a more accurate taxonomic identification due to the degradation of morphological characteristics required for the use of dichotomous keys.

These severe environmental factors highlight the complexity of the local ecosystem and the influence of wildfires on vector population dynamics. The composition of Culicidae communities showed variations influenced by the seasonal period, reflecting the complex interaction among physical and abiotic factors, competition, and predation, which shape patterns of species occurrence and distribution. These factors are closely linked to biological and evolutionary processes that affect mosquito population dynamics in the Amazon region [82,83]. The species *Cq. venezuelensis* and *Cq. arribalzagae* were recorded throughout all sampling periods, indicating their broad environmental adaptability. In contrast, species exclusive to the rainy season, such as *Tr. digitatum*, *Sa. amazonicus*, *Ps. ferox*, *Ps. albipes*, *Ma. indubitans*, *Li. durhamii*, *Ae. serratus*, and *Cx. pedroi,* reflect a direct dependence on the hydrological cycle for their development. During the transitional period, species such as *Ur. geometrica*, *Ur. ditaeniota*, *Sa. tarsopus*, *Ma. pseudotitillans*, *Ha. albomaculatus*, and several species of the genera *Culex* and *Anopheles* were observed, evidencing adaptations to more varied and less extreme environmental conditions.

Another species, although less abundant, was recorded in this study, mainly in higher strata. The genus *Sabethes* Robineau-Desvoidy is commonly found in wild environments, typically in the forest canopy [30]. The species *Sa. chloropterus* identified in this study is an important vector of YFV in areas where humans and primates coexist [84]. It is necessary to emphasize the importance of the genus *Sabethes* in maintaining the circulation of this virus, acting as a maintainer of epizootics during the dry season, especially in the absence of the primary vectors of the genus *Haemagogus* [85].

In the present study, the species *Hg. janthinomys*, although recorded in low abundance, holds great epidemiological importance. It plays a central role in the sylvatic cycle of arboviruses, particularly in the higher strata of the forest, being associated with epizootics caused by YFV and MAYV in non-human primates and humans. Together with *Hg. leucocelaenus*, *Hg. janthinomys* was involved in the most recent major yellow fever epidemic in Brazil, which occurred between 2016 and 2018 and resulted in 2058 confirmed cases and 689 deaths [86].

The genus *Anopheles* is important in the transmission of *Plasmodium* spp., the causative agent of malaria, which is endemic in the Amazon region. However, this genus has also been associated with the transmission of the arbovirus *Alphavirus onyong*, responsible for epidemics in Uganda, Tanzania, Kenya, Malawi, Senegal, and the Democratic Republic of Congo. Several other arboviruses have already been detected in *Anopheles* pools, distributed across the families Flaviviridae and Peribunyaviridae and within the genera *Alphavirus*, *Orbivirus*, and *Phlebovirus*, among others [87].

In the present study, 164 specimens of the genus *Anopheles* were recorded, including *An. peryassui* (*n* = 38; 0.03%), *An. aquasalis*, and *An. triannulatus*, both represented by only one specimen each. *Anopheles peryassui* typically breeds in natural aquatic habitats such as ponds and reservoirs rich in organic matter [88]. Adults generally exhibit zoophilic behavior, feeding preferentially on other mammals rather than humans, and exhibit crepuscular or nocturnal activity patterns [89,90]. Although not recognized as a malaria vector, some specimens have been found naturally infected with *Plasmodium* spp. in the Amazon region [91].

*Anopheles aquasalis* is epidemiologically significant in the Amazon, particularly due to its established role as an efficient vector of *Plasmodium vivax*. It is zoophilic, crepuscular, and predominantly exophilic, especially at high population densities. Reproduction occurs mainly in stagnant or slow-moving water, including temporary or semi-permanent habitats such as seasonally flooded lowlands influenced by tidal movements, as well as puddles and ditches formed during the rainy season in saline or brackish soils [25,30].

*Anopheles triannulatus*, in turn, displays distinct bioecological traits, with immature stages developing in lagoons rich in floating vegetation, such as water hyacinths (*Eichhornia*), water lettuce (*Pistia*), and other plants [25]. It is primarily zoophilic, exophilic, and crepuscular, occasionally biting humans. While not considered a primary malaria vector in Brazil, it has been found naturally infected with *Plasmodium* spp. in Colombia [92].

Although only three species were recorded in this study, previous research indicates considerably higher diversity across the state of Amapá. For example, Galeno (2016), in Mazagão [93], reported seven species; Barbosa et al. (2016) recorded 1330 specimens across nine species [94]; Póvoa et al. (2001), in Serra do Navio, identified 3035 specimens representing 15 species [95]; and Fonseca et al. (2021) documented 3810 specimens among 12 species [96]. Notably, these studies employed various sampling methods, including Shannon-type traps and protected human baits during nighttime collections, which coincide with the peak activity periods of most *Anopheles* species and may have influenced species composition and collection rates. In comparison, the present study was limited by collection methods focused primarily on diurnal periods, potentially underestimating species richness and overall diversity.

The maintenance and dynamics of *Anopheles* populations are closely linked to the availability and characteristics of their breeding habitats. Natural habitats, such as ponds, lakes, and seasonally flooded lowlands, provide essential conditions for oviposition and larval development, supporting the persistence of local populations and maintaining important ecological interactions with other species [97]. In addition, artificial habitats, particularly fishponds and other man-made water bodies, can play a significant role in *Anopheles* population dynamics, often increasing larval abundance and providing alternative breeding sites in areas impacted by human activity. For example, Reis et al. (2015), in the state of Acre, observed that artificial fishponds contained four times more *Anopheles* larvae than natural water bodies, highlighting the importance of these habitats in vector proliferation [98].

Notably, these studies employed various sampling methods, including Shannon-type traps and protected human baits during nighttime collections, in addition to the collection of immature stages conducted during the day. The nighttime methods coincide with the peak activity periods of most *Anopheles* species and likely influence species composition and collection rates. In comparison, the present study focused primarily on natural habitats, employing daytime sampling methods (PHA) and CDC traps without additional attractants during the dusk/night period, which may have resulted in an underestimation of species richness and overall diversity.

Building on these broader regional patterns, the present study represents a pioneering effort to encompass the rainy, dry, and intermediary seasons in the state of Amapá, providing a detailed perspective on the remarkable mosquito biodiversity of this Amazonian frontier. By collecting data across contrasting climatic periods, the study contributes to a more comprehensive ecological understanding and provides a foundation for assessing the influence of human activities on local mosquito communities. Nevertheless, important limitations remain, including the restricted sampling duration and limited spatial coverage, highlighting the need for future studies with expanded temporal and geographical scope to more fully characterize the diversity and dynamics of these vector populations.

## 5. Conclusions

This study, conducted at Vila Maranhão farm in the rural area of Mazagão-AP, identified 38 mosquito species distributed across 15 genera. The rainy and intermediary periods exhibited greater abundance and diversity of species compared to the dry season, during which *Cq.* (*Rhy.*) *venezuelensis* was eudominant. Smoke resulting from human activities was likely the crucial factor interfering with species abundance and richness during the dry period.

Both CDC and PHA traps captured higher numbers of *Cq.* (*Rhy.*) *venezuelensis*, highlighting the importance of complementary methods, such as Shannon traps, to achieve greater sampling representativeness. The interpolated and extrapolated curves suggest sufficient sampling effort for the intermediary and dry periods but indicate greater uncertainty during the rainy season.

The presence of epidemiologically relevant species involved in arbovirus transmission highlights the need for further entomofaunal studies in the state and underscores the potential risk to public health. Viral isolation and metagenomic studies are required to better understand mosquito–virus–animal interactions, enabling the early identification of potential pathogens circulating in forest environments that may spill over to humans.

## Figures and Tables

**Table 1:** *Culicidae* species collected during the rainy, intermediary, and dry periods using the PHA and CDC methods, both at ground and canopy levels.

Species	Rainy				Intermediary				Dry					
	PHA		CDC		PHA		CDC		PHA		CDC			
	Gr	Ca	Gr	Ca	Gr	Ca	Gr	Ca	Gr	Ca	Gr	Ca	Total	Fre. (%)
*Ad.* (*Ady.*) *squamipennis*	0	0	28	4	0	0	5	3	0	0	0	0	40	1.14
*Ae.* (*Och.*) *serratus*	5	1	1	0	0	0	0	0	0	0	0	0	7	0.20
*An.* (*Ano.*) *peryassui*	0	0	0	0	0	0	33	5	0	0	0	0	38	1.09
*An.* (*Ano.*) spp.	0	0	0	0	0	0	39	7	0	0	0	0	46	1.31
*An.* (*Nys.*) *aquasalis*	0	0	0	0	0	0	0	0	1	0	0	0	1	0.03
*An.* (*Nys.*) *triannulatus*	0	0	0	0	0	0	1	0	0	0	0	0	1	0.03
*An.* (*Nys.*) spp.	7	0	0	0	8	1	39	46	0	0	0	0	101	2.89
*Anopheles* spp.	0	0	0	0	0	0	17	0	0	0	0	0	17	0.49
*Cq.* (*Rhy.*) *albicosta*	2	1	9	6	2	3	130	55	0	0	0	0	208	5.94
*Cq.* (*Rhy.*) *arribalzagae*	1	0	0	0	1	0	2	1	9	1	0	0	15	0.43
*Cq.* (*Rhy.*) *juxtamansonia*	0	0	0	0	0	1	1	0	0	0	0	0	2	0.06
*Cq.* (*Rhy.*) *nigricans*	0	0	2	1	2	0	2	0	0	0	0	0	7	0.20
*Cq.* (*Rhy.*) spp.	20	0	27	40	89	21	327	90	0	0	0	2	616	17.60
*Cq.* (*Rhy.*) *venezuelensis*	15	3	143	150	10	0	195	110	0	0	7	0	633	18.09
*Cx.* (*Cux.*) spp.	5	0	22	6	1	0	13	5	0	0	4	0	56	1.60
*Cx.* (*Mel.*) *ensiformis*	0	0	0	0	0	0	1	0	0	0	0	0	1	0.03
*Cx.* (*Mel.*) group *atratus*	0	0	27	15	0	0	79	36	0	0	0	0	157	4.49
*Cx.* (*Mel.*) *pedroi*	0	0	8	1	0	0	0	0	0	0	0	0	9	0.26
*Cx.* (*Mel.*) *portesi*	0	1	0	9	0	0	110	26	0	0	0	0	146	4.17
*Cx.* (*Mel.*) *longisetosus*	0	0	0	0	0	0	0	2	0	0	0	0	2	0.06
*Cx.* (*Mel.*) spp.	0	0	13	34	4	0	212	67	0	0	60	35	425	12.14
*Cx.* (*Mel.*) *spissipes*	6	0	37	23	0	0	53	11	0	0	0	0	130	3.71
*Cx.* (*Mel.*) *vomerifer*	0	0	0	0	0	0	4	1	0	0	1	0	6	0.17
*Hg.* (*Hag.*) *albomaculatus*	0	0	0	0	2	0	0	0	0	0	0	0	2	0.06
*Hg.* (*Hag.*) *janthinomys*	0	1	1	0	0	0	2	2	0	0	0	0	6	0.17
*Hg.* (*Hag.*) sp.	0	0	0	0	0	1	0	0	0	0	0	0	1	0.03
*Li. durhamii*	1	1	0	0	0	0	0	0	0	0	0	0	2	0.06
*Limatus* spp.	6	0	0	0	6	0	0	0	0	0	0	0	12	0.34
*Ma.* (*Man.*) *indubitans*	0	0	5	3	0	0	0	0	0	0	0	0	8	0.23
*Ma.* (*Man.*) *pseudotitillans*	0	0	0	0	0	0	3	0	0	0	0	0	3	0.09
*Ma.* (*Man.*) spp.	0	1	267	70	0	0	0	6	0	0	2	0	346	9.89
*Ma.* (*Man.*) *titillans*	1	1	0	0	24	83	67	27	0	0	0	0	203	5.80
*Ps.* (*Jan.*) *albipes*	0	0	1	1	0	0	0	0	0	0	0	0	2	0.06
*Ps.* (*Jan.*) *ferox*	11	1	0	0	0	0	0	0	0	0	0	0	12	0.34
*Ps.* (*Jan.*) sp.	2	1	0	0	0	0	0	0	0	0	0	0	3	0.09
*Sa.* (*Sab.*) *tarsopus*	0	0	0	0	0	7	0	0	0	0	0	0	7	0.20
*Sa. (Sab.*) *amazonicus*	0	2	0	0	0	0	0	0	0	0	0	0	2	0.06
*Sa.* (*Sab.*) *belisarioi*	0	2	0	0	0	2	0	0	0	0	0	0	4	0.11
*Sa.* (*Sab.*) *cyaneus*	0	2	0	0	0	1	0	0	0	0	0	0	3	0.09
*Sa.* (*Sab.*) *quasicyaneus*	0	1	0	0	0	1	0	0	0	0	0	0	2	0.06
*Sa.* (*Sbo.*) *chloropterus*	1	2	0	0	0	3	0	0	0	0	0	0	6	0.17
*Sabethes* spp.	0	0	0	0	0	4	0	0	0	0	0	0	4	0.11
*Tr.* (*Trc.*) *digitatum*	1	1	0	0	0	0	0	0	0	0	0	0	2	0.06
*Ur.* (*Ura.*) *ditaeniota*	0	0	0	0	0	0	0	1	0	0	0	0	1	0.03
*Ur.* (*Ura.*) *geometrica*	0	0	0	0	0	0	11	1	0	0	0	0	12	0.34
*Uranotaenia* (*Ura.*) spp.	0	0	0	1	0	0	35	0	0	0	102	24	162	4.63
*Wy. negrensis/occulta*	2	0	0	0	0	0	0	0	0	0	0	0	2	0.06
*Wyeomyia* spp.	16	0	0	0	7	0	4	1	0	0	1	0	29	0.83
Abundance	1079				2172				249				3500	

Legend: Fre. = relative frequency; D > 10% = eudominant; D > 5 < 10% = dominant; D > 2% < 5% = subdominant; D 1 < 2% = possible; and D < 1% = rare; Gr (Ground); Ca (Canopy); PHA (Protected Human Attraction); and CDC (Centers for Disease Control and Prevention).

## Data Availability

The original contributions presented in this study are included within this article/Supplementary Materials. Further inquiries can be directed to the corresponding author.

## Supplementary Materials

The following supporting information can be downloaded at: https://www.mdpi.com/article/10.3390/insects16101036/s1, Tabel S1: Meteorological variables recorded during the rainy period, with values representing the daily averages of relative humidity (%), temperature (°C), and precipitation (mm), Table S2: Meteorological variables recorded during the dry period, with values representing the daily averages of relative humidity (%), temperature (°C), and precipitation (mm), and Table S3: Meteorological variables recorded during the intermediary period, with values representing the daily averages of relative humidity (%), temperature (°C), and precipitation (mm).
