# Supplementary material for: Culicidae Fauna (Diptera: Culicomorpha) of the Municipality of Mazagão, Amapá, in the Brazilian Amazon
Source: Insects. 2025 Oct 9;16(10):1036. doi: 10.3390/insects16101036 (PMC12565007; doi:10.3390/insects16101036)
Supplement: Supplementary file 1 [file insects-16-01036-s001.zip › insects-3877201-supplementary.pdf]

Supplementary Table S1. Meteorological variables recorded during the rainy period, with values representing the daily averages of relative humidity (%), temperature (°C), and precipitation (mm).

Rainy			
**Date**	**Humidity (%)**	**Temperature (ºC)**	**Precipitations (mm)**
18/04/2024	89.5	27.1	3.0
19/04/2024	87.5	27.4	0.1
20/04/2024	83.5	28.4	6.8
21/04/2024	90.0	27.2	0
22/05/2024	95	26.6	11.7

Supplementary Table S2. Meteorological variables recorded during the dry period, with values representing the daily averages of relative humidity (%), temperature (°C), and precipitation (mm).

Dry			
**Date**	**Humidity (%)**	**Temperature (ºC)**	**Precipitations (mm)**
13/11/2023	62.5	32.8	0
14/11/2023	60.5	32.8	0
15/11/2023	58.5	31.8	0
16/11/2023	66.5	33.1	0
17/11/2023	63.5	31.7	0

Supplementary Table S3. Meteorological variables recorded during the intermediary period, with values representing the daily averages of relative humidity (%), temperature (°C), and precipitation (mm).

Intermediary			
**Date**	**Humidity (%)**	**Temperature (ºC)**	**Precipitations (mm)**
21/07/2023	88.9	28.1	0
22/07/2023	85.3	27.1	0
23/07/2023	87.1	29.5	0.2
24/07/2023	89.0	29.2	0
25/07/2023	86.8	27.7	6.6
